# Hindu Spiritual Music for Perioperative Anxiolysis and Stress Modulation: An Open-Label, Randomized Comparative Trial in Lower Limb Surgery Patients

**DOI:** 10.7759/cureus.80642

**Published:** 2025-03-16

**Authors:** Anshul Jain, Brijendra Verma, Pankaj Saunakiya, Saurabh Agarwal, Pushpendra Agarwal, Paras Gupta, Charu Thakur

**Affiliations:** 1 Anesthesiology, Maharani Laxmi Bai Medical College, Jhansi, IND; 2 Surgery, Maharani Laxmi Bai Medical College, Jhansi, IND; 3 Orthopedics, Maharani Laxmi Bai Medical College, Jhansi, IND; 4 Surgery, Rajkiya Medical College, Jalaun, IND; 5 Orthopedics, Rajkiya Medical College, Jalaun, IND

**Keywords:** hindu spiritual music, patient satisfaction, perioperative anxiety, regional anesthesia, serum cortisol, spiritual intervention, stress response

## Abstract

Background

Perioperative anxiety is a common concern in patients undergoing regional anesthesia, as they remain conscious during surgery. Various non-pharmacological interventions, including music therapy, have been explored to reduce anxiety and stress response. Spiritual interventions, particularly religious music, have been shown to provide emotional stability and enhance coping mechanisms during stressful situations.

Objective

This study aimed to evaluate the impact of Hindu religious music compared to patient-selected instrumental music on intraoperative anxiety, stress response, and patient satisfaction in individuals undergoing lower limb surgery under regional anesthesia.

Methods

This prospective, open-label, randomized controlled trial was conducted at Maharani Laxmi Bai Medical College, a tertiary care teaching hospital in Jhansi, India, after obtaining approval from the Institutional Ethics Committee (Certificate No. 5208/IEC/I/2022-2023) and registration with the Clinical Trials Registry of India (CTRI/2024/01/062006, registered on January 30, 2024). The trial included 150 ASA class 1 or 2 patients undergoing elective lower limb surgery under regional anesthesia. Participants were randomly assigned to two groups: Group A (Hindu spiritual music) and Group B (patient-preferred instrumental music). Music was played intraoperatively via noise-canceling headphones. The primary outcomes were intraoperative anxiety (measured using the Visual Analog Scale for Anxiety (VAS-A)) and serum cortisol levels as a biochemical marker of stress. Secondary outcomes included hemodynamic parameters, additional analgesic requirements, incidence of nausea and vomiting, and patient satisfaction.

Results

Group A had significantly lower intraoperative VAS-A scores compared to Group B (2.13 ± 0.91 vs. 3.41 ± 1.12; p = 0.01). Serum cortisol levels were also significantly lower in Group A at the end of surgery (28.54 ± 6.11 vs. 32.50 ± 8.82 µg/dL; p = 0.001) and remained lower on the first postoperative day (p = 0.001). The incidence of postoperative nausea and vomiting was significantly lower in Group A (6.8% vs. 17.5%; p = 0.028). Patient satisfaction was also higher in the Hindu spiritual music group (p = 0.03).

Conclusion

Hindu spiritual music reduced perioperative anxiety, attenuated the stress response, and decreased postoperative nausea and vomiting compared to non-spiritual instrumental music. It should be considered a non-pharmacological intervention in the perioperative period to alleviate anxiety and improve patient comfort. However, the study's findings may have limited generalizability to non-Hindu patients. Future research should explore whether similar benefits are observed in religious individuals listening to music of their own faith and cultural background.

## Introduction

Anxiety and pain are common challenges faced by patients undergoing lower limb surgeries, particularly when performed under regional anesthesia. These procedures expose patients to intraoperative conversations and ambient noises, which may heighten their anxiety levels and potentially lead to physiological and psychological complications. Effective management of this anxiety is crucial, as heightened preoperative stress is associated with alterations in the pharmacokinetics of anesthetic agents and can adversely affect recovery​ [1,2].

Traditionally, pharmacological approaches such as opioids and benzodiazepines have been employed to manage perioperative anxiety and pain [3,4]. However, these medications are known for their sedative, emetic, and dose-dependent side effects, including respiratory and central nervous system depression​ [3].

Spiritual interventions have been widely explored for their role in reducing stress and anxiety, particularly in medical and high-stress environments [5,6]. Spirituality provides a sense of comfort, resilience, and emotional regulation, helping individuals cope with fear and uncertainty. Various spiritual practices, including prayer, meditation, and chanting, have been studied for their anxiolytic effects, with research suggesting their ability to modulate neuroendocrine and autonomic responses. Among different forms of spiritual interventions, music holds a unique place, offering a non-invasive and easily accessible method to induce relaxation and emotional well-being. Spiritual music, rooted in faith and cultural beliefs, has been shown to influence mood, cognitive processing, and physiological responses associated with stress. However, while music as an intervention has been extensively studied, limited research has focused on spiritual music as a distinct therapeutic intervention [7,8].

Individuals who identify as religious (theistic) often seek spiritual or religious support during stressful situations. This is observed across various faiths - Christians remember Jesus, Muslims invoke Allah, and Hindus chant prayers or mantras to their deities in times of distress. Based on this, we hypothesize that religious music may have a greater impact on anxiety and stress relief compared to non-religious music among religious individuals. To test this hypothesis, a randomized controlled trial (RCT) was conducted with the primary aim of evaluating the effectiveness of Hindu religious music in reducing perioperative anxiety and stress response compared to non-religious music. The secondary objectives included assessing physiological outcomes to determine the broader hemodynamic effects of religious music, evaluating patient satisfaction to understand its impact on the overall perioperative experience, and exploring personalized music therapy to recommend tailored music selections based on patients' cultural and religious backgrounds. This study assesses the impact of religious music as a spiritual intervention. While the study focuses on Hindu theistic patients, the findings offer insights into the use of spiritual music in other religious populations.

## Materials and methods

This study was designed as a prospective, open-label, randomized comparative trial. The study protocol and procedure were approved by the Institutional Ethics Committee (Certificate No. 5208/IEC/I/2022-2023 Dated 17/11/2023) and adhered to the principles outlined in the Declaration of Helsinki (2013) and Good Clinical Practice guidelines. Written informed consent was obtained from all participants prior to enrollment. The study was prospectively registered with the Clinical Trials Registry-India (CTRI/2024/01/062006 (registered on January 30, 2024)) and followed the CONSORT checklist for manuscript preparation.

Study population

A total of 150 Hindu theistic patients, aged between 18 and 60 years, with an American Society of Anesthesiologists (ASA) physical status classification I or II, scheduled for elective lower limb surgery under regional anesthesia, were recruited for this study. The expected surgical duration for all cases was two hours or less. All participants underwent screening during the pre-anesthetic evaluation to confirm eligibility. Patients were excluded from the study if they had a diagnosis of diabetes mellitus, pregnancy, hearing disabilities, a preoperative Ramsay Sedation Score greater than 3, were on psychiatric medications or sedatives, or systemic opioids.

Conduct of study

The study was conducted at Maharani Laxmi Bai Medical College, a tertiary care teaching hospital in Jhansi, India, equipped with modern operation theaters and an in-house pathology laboratory. Participants meeting the study criteria were screened during the pre-anesthetic evaluation. Computer-based randomization was employed to allocate participants into two groups. All participants were informed of the study procedures and provided written consent prior to enrollment.

On the day of surgery, patients were re-evaluated in the preoperative room. A blood sample was taken to measure preoperative serum cortisol levels before the initiation of the music intervention. Baseline parameters, including heart rate, systolic blood pressure (SBP), diastolic blood pressure (DBP), and Visual Analog Scale for Anxiety (VAS-A) scores, were recorded [9]. Patients were also introduced to the Visual Analog Scale (VAS) for analgesia, which uses a 1-10 scale [10]. They were instructed that if their VAS score exceeded 2 during surgery, additional analgesics would be administered to manage pain. Injection ondansetron 4 mg IV was administered to all patients in the preoperative period as standard prophylaxis for intraoperative nausea.

The music intervention was initiated before shifting the patient to the operating theater, briefly paused during the administration of the neuraxial block, and resumed afterward. Upon transfer to the operating theater, standard monitors including electrocardiography, pulse oximetry, and noninvasive blood pressure monitoring were applied. Venous access was secured for fluid administration. Under sterile conditions, neuraxial blockade was administered at the L3/L4 interspace using a 25-gauge pencil-point spinal needle. A dose of 2.5 to 3 mL (0.3 mL/kg) of hyperbaric 0.5% bupivacaine was injected while the patient was in a seated position. For patients undergoing procedures expected to last longer than 120 minutes or those with fractures, an epidural catheter was placed for extended pain control. Once the desired sensory block level was confirmed, the patient was positioned appropriately for surgery. Patients who experienced inadequate block height or required supplementary anesthesia were excluded from the study.

Throughout the surgery, continuous intraoperative monitoring of heart rate, respiratory rate, oxygen saturation (SpO₂), and blood pressure was maintained. Intraoperative VAS-A and VAS analgesia scores were recorded, along with any need for sedatives or rescue analgesics. In patients with subarachnoid block, rescue analgesia was provided with intravenous tramadol (administered at 1 mg/kg and rounded off to the nearest 10 mg). In patients with an epidural catheter, a bolus of 6 mL of 0.125% bupivacaine was administered via the catheter for additional pain control. At the conclusion of the surgery, the music was stopped, and patients were transferred to the postoperative care unit (PACU). Postoperative monitoring continued, with vital signs and hemodynamic parameters recorded for further evaluation.

Intervention

Participants meeting the study criteria were asked to report 10 of their favorite movie songs during a pre-study visit the night before surgery. In addition, they were provided with a list of 10 commonly heard Hindu religious songs and given the option to remove two songs from the list.

On the day of surgery, the participants were randomized into two groups using computer-generated allocation in the preoperative room: *Group A*: Participants listened to common hindu religious songs through headphones during the intraoperative period. *Group B:* Participants listened to instrumental music created from their personally provided list of favorite movie songs.

In both groups, the selected music tracks were arranged into loops of eight tracks. For Group A, a loop of eight common religious songs was played (Appendix A), while for Group B, a loop of eight instrumental tracks derived from the patients' chosen songs was created. The music was sourced from Apple Music, ensuring the highest available audio quality. The selected tracks were downloaded in the best possible quality, with a minimum bitrate of 320 kbps, to maintain optimal sound fidelity. The music was played through noise-cancellation headphones in both groups (Appendix B).

Outcome parameter

The primary outcomes of the study included the VAS-A score, which was used to assess anxiety in the perioperative period, and the stress response, evaluated by measuring serum cortisol levels immediately after the end of surgery and again on the first postoperative day. The secondary outcomes comprised patient vitals, including heart rate, blood pressure, and oxygen saturation, as well as additional analgesic requirements recorded as the administration of rescue analgesics for pain control during surgery. The incidence of nausea and vomiting was assessed both intraoperatively and postoperatively. In addition, patient satisfaction was evaluated 24 hours postoperatively using a five-point Likert scale, ranging from "very satisfied" to "very dissatisfied."

Measurements and data handling

Baseline vitals, including heart rate, systolic blood pressure (SBP), diastolic blood pressure (DBP), and Visual Analog Scale for Anxiety (VAS-A) scores, were measured in the preoperative room. Serum cortisol level was also measured at this time using electrochemiluminescence immunoassay.

The VAS-A score was assessed twice during the intraoperative period: first, 30 minutes after the skin incision, and second, immediately upon completion of the procedure to evaluate changes in anxiety levels. Serum cortisol levels were measured immediately after the end of surgery and repeated on the first postoperative day. The number of patients who requested the music intervention to be stopped or headphones to be removed during surgery was also documented. Nausea and vomiting events were documented during surgery and within the first 24 hours postoperatively. The need for rescue antiemetics was also recorded.

Patient satisfaction was evaluated on first postoperative day visit. Serum cortisol levels were measured again on the first and third postoperative days.

Sample size

The sample size for this study was calculated based on a comparison of two independent groups with a continuous outcome (anxiety scores). Assuming a moderate effect size (Cohen’s d = 0.5, d = 0.5), a power of 90% (Zβ = 1.28, Zβ ​= 1.28), and an alpha error of 5% (Zα/2 = 1.96, Zα/2 ​= 1.96), the required sample size per group was calculated using the formula for two independent groups. Based on prior studies and an equal allocation ratio of 1:1 between groups, the standard deviation (σ) of anxiety scores was estimated to be 0.86. The formula used for the calculation is:

n= [2.(Z_α/2_+ Z_β_)^2^.σ^2^]/d^2^

Based on these parameters, the calculated sample size was 134 participants. To account for potential dropouts and technical failures, the sample size was increased to 150 participants, with 75 participants allocated to each group.

Statistical analysis

Continuous variables were presented as mean ± standard deviation (SD), medians, ranges (minimum to maximum), and 95% confidence intervals (CIs). Categorical data were expressed as frequencies and percentages. Group comparisons for categorical variables were performed using the Chi-square test or Fisher’s exact test, as appropriate. For continuous variables, an independent samples t-test (Student's t-test) was used for normally distributed data, while the Mann-Whitney U test was applied for non-normally distributed data. All statistical analyses were conducted using IBM SPSS Statistics for Windows, Version 29.0 (released 2023, IBM Corp., Armonk, NY). A p-value of less than 0.05 was considered statistically significant.

## Results

**Figure 1:**
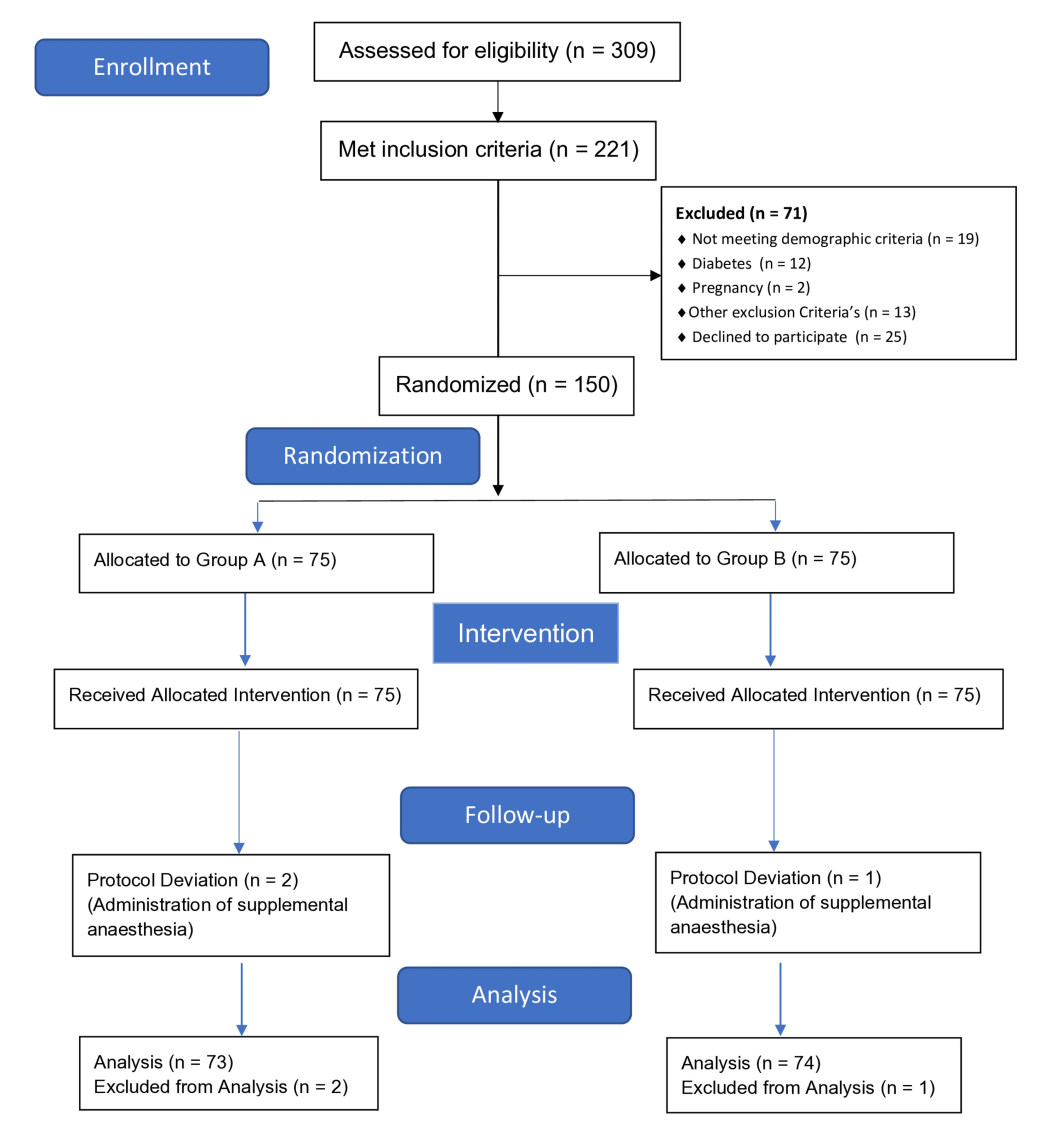
CONSORT flow Diagram

The trial was conducted between February 2024 and December 2024. The first patient was enrolled on February 4, 2024. During the recruitment period, 309 patients were screened for eligibility. Of these, 221 patients met the inclusion criteria, and 71 patients were excluded based on predefined exclusion criteria. Recruitment ceased after the target sample size was reached, with the final patient enrolled on November 18, 2024. A total of 150 patients were randomized into two groups, as illustrated in the CONSORT flow diagram (Figure 1).

The demographic and baseline characteristics of the participants are presented in Table 1. The mean age of patients in Group A was 41.03 ± 8.80 years, while in Group B, it was 44.66 ± 9.71 years, with no statistically significant difference between the groups (p = 0.07). In addition, there were no significant differences between the groups in other baseline parameters, including American Society of Anesthesiologists (ASA) physical status, body mass index (BMI), baseline hemodynamic parameters, serum cortisol levels, and Visual Analog Scale for Anxiety (VAS-A) scores (Table 1).

**Table 1:** Baseline demographic, clinical, and physiological characteristics of the study participants SD: standard deviation

Parameters	Group A (N = 75)	Group B (N = 75)	p-value
Age (in years) Mean ± SD	41.03 ± 8.80	44.66 ± 9.71	0.070
Weight in Kg (Mean ± SD)	70.45 ± 5.90	68.89 ± 6.30	0.089
Height in cm (Mean ± SD)	162.84 ± 5.12	164.23 ± 4.97	0.070
Male:female ratio	53 : 22	51 : 24	0.08
Patient clinical speciality			
Orthopedic patients (%)	81.33% (N = 61)	80.00% (N = 60)	0.65
General surgery patients (%)	18.67% (N = 14)	20.00% (N = 15)	0.65
Mode of anesthesia:			
Subarachnoid block (%)	62.7% (N = 47)	59.3% (N=44)	0.09
Subarachnoid block with epidural (%)	37.3% (N = 28)	40.7% (N = 31)	0.09
ASA status			
ASA status I (%)	53.33% (N = 40)	54.67% (N = 41)	0.42
ASA status II (%)	46.67% (N = 35)	45.33% (N = 34)	0.42
Baseline vitals			
Heart rate (bpm) Mean ± SD	91.13 ± 6.93	89.87 ± 8.12	0.31
SBP (mm of Hg) Mean ± SD	124.28 ± 11.56	127.81 ± 10.30	0.053
DBP (mm of Hg) Mean ± SD	83.13 ± 6.91	84.83 ± 7.23	0.14
*VAS-A score* (Mean ± SD)	4.2 ± 1.3	4.4 ± 1.4	0.08
*Serum cortisol level* (mcg/dL)	22.19 ± 4.18	21.68 ± 6.12	0.55

Of the initial 150 patients, two patients from Group A and one patient from Group B required additional anesthesia, leading to their exclusion from the final data analysis. This resulted in a final sample size of 73 for Group A and 74 for Group B. During the surgery, two patients in Group A and five patients in Group B requested that the music intervention be discontinued. This resulted in compliance rates of 97.3% (71/73) for Group A and 93.2% (69/74) for Group B. The difference was not found to be statistically significant (p = 0.2).

The mean intraoperative VAS-A score was significantly lower in Group A than in Group B (2.13 ± 0.91 vs. 3.41 ± 1.12; p = 0.01). The VAS-A score at the end of the procedure was also significantly lower in Group A (1.89 ± 0.85) compared to Group B (2.50 ± 0.97; p = 0.03). The mean serum cortisol level (mcg/dL) at the end of surgery was significantly lower in Group A compared to Group B (28.54 ± 6.11 vs. 32.50 ± 8.82; p = 0.01). This significant difference in serum cortisol persisted on the first postoperative day as well (Table 2).

**Table 2:** Comparison of anxiety, stress response, and additional analgesic requirement between Hindu spiritual music and non-spiritual music groups #: statistically significant

Parameter	Group A (N = 73)	Group B (N = 74)	P-value
VAS-A score (mean ± SD)			
Baseline	5.62 ± 1.02	5.94 ± 1.35	0.62
Intraoperative	2.13 ± 0.91	3.41 ± 1.12	0.01^#^
End of procedure	1.89 ± 0.85	2.50 ± 0.97	0.03^#^
Serum cortisol (mean ± SD)			
Baseline	22.19 ± 4.18	21.68 ± 6.12	0.56
End of surgery (mcg/dL)	28.54 ± 6.11	32.50 ± 8.82	0.001^#^
1st post-op day (mcg/dL)	29.02 ± 7.01	35.91 ± 8.10	0.001^#^
3rd post-op day ( mcg/dL)	32.50 ± 6.95	37.35 ± 9.90	0.001^#^
Additional analgesic requirement (%)	9 (12.33%)	15 (20.27%)	0.06

**Figure 2:**
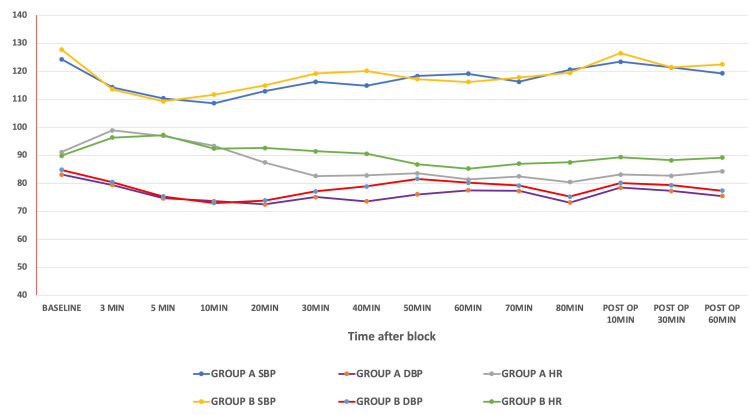
Comparison of perioperative hemodynamics between groups SBP: systolic blood pressure; DBP: diastolic blood pressure; HR: heart rate

The requirement for additional intraoperative analgesics was lower in Group A, with nine patients (12.33%) requiring rescue analgesia compared to 15 patients (20.27%) in Group B (odds ratio 0.38 (95% CI: 0.14-1.05); p = 0.06). However, this difference was not statistically significant. Similarly, no significant difference was observed in systolic and diastolic blood pressure between the two groups during the intraoperative period. The mean heart rate was significantly lower in Group A compared to Group B (86.51 ± 8.86 bpm vs. 89.28 ± 6.19 bpm; p = 0.03) (Figure 2). The incidence of intraoperative nausea and vomiting was comparable between the two groups (5.5%, n = 4 in Group A vs. 6.8%, n = 5 in Group B; p = 0.76). In the postoperative period, the incidence of nausea and vomiting was significantly higher in Group B (17.5%, n = 13) compared to Group A (6.84%, n = 5; p = 0.028) (Table 3).

**Table 3:** Incidence of adverse events in the study groups

Side effects	Group A (N = 73)	Group B (N = 74)	P- value
Intraoperative: number of patients, (%)			
Bradycardia	5 (6.84%)	4.05% (N = 3)	0.49
Hypotension	7 (11.68%)	10 (13.5%)	0.60
Shivering	6 (8.21%)	7 (9.45%)	^1^
Nausea	4 (5.47%)	5 (8.10%)	1
Dyspnoea	0	0	NA
Pruritis	12.32% (9)	10.81%(8)	0.80
Postoperative : number of patients (%)			
Nausea/vomiting	5 (6.84%)	13 (17.56)	0.028
PDPH	5 (6.84%)	7 (9.45%)	0.765

The distribution of patient satisfaction levels showed a statistically significant difference between Group A and Group B, with higher satisfaction in Group A (p = 0.031, Mann-Whitney U test) (Table 4).

**Table 4:** Group-wise distribution of patient satisfaction levels

Satisfaction level	Group A (n = 73)	Group B (n = 74)
Very satisfied	48 (65.8%)	21 (28.37%)
Satisfied	20 (27.4%)	38 (51.4%)
Neutral	3 (4.1%)	10 (13.5%)
Dissatisfied	2 (2.7%)	4 (5.4%)
Highly dissatisfied	0 (0%)	1 (1.35%)

## Discussion

In the present study, the tested hypothesis was found to be true, with Hindu spiritual music proving to be more effective in reducing intraoperative anxiety and stress response than non-spiritual, subject-selected instrumental music. Spirituality has been widely researched as an intervention for mental well-being, with numerous studies highlighting its positive role in coping with stress, anxiety, and fear. Hinduism, one of the world’s oldest religions, has deeply ingrained spiritual traditions, with practices such as chanting mantras and invoking deities being common responses to stress and uncertainty [11]. This is particularly evident in preoperative settings, where many patients are observed chanting religious prayers or mantras before entering the operating room or undertaking significant life events. Despite Hinduism being one of the world's oldest religions, research on Hindu spiritual interventions in healthcare remains limited. Several studies have explored the role of spirituality in medical settings. Hosseini et al. found that a structured religious intervention significantly reduced preoperative anxiety in patients undergoing major surgery​[12]. Another study by Kalkhoran et al. observed a correlation between religiosity and reduced preoperative anxiety, although statistical significance was not achieved​ [13]. In addition, research suggests that faith-based coping strategies provide emotional stability and psychological comfort, making them valuable tools for anxiety management in high-stress environments [14,15]. Music could serve as an easily accessible spiritual intervention in perioperative settings.

Since ancient times, music has been used as both an adjuvant and a primary therapeutic modality. Historical records suggest that Imhotep, one of the greatest physicians of Ancient Egypt, employed music therapy and even established a sanatorium dedicated to its practice [16]. The use of music for pain relief has also been documented for centuries, emphasizing its longstanding role in holistic healing approaches. Although the precise mechanism underlying the central effects of music remains unclear, some studies suggest that it may act as a positive distraction, influence the cognitive processing of pain, and stimulate the release of beta-endorphins, leading to enhanced emotional responses and mood improvement [17,18].

In our study, we used the VAS-A to assess anxiety levels. The VAS-A is a well-validated and easily interpretable tool for measuring anxiety, with strong correlation to other standardized anxiety scales [9,19]. Its role in perioperative settings has been well established, with previous studies demonstrating its effectiveness in assessing anxiety levels in surgical patients. To minimize observer bias, participants in non-religious music groups selected their preferred music based on their individual preference. We observed a greater reduction in VAS-A scores in the Hindu spiritual music group, aligning with the findings of Premraj et al., who also reported a significant reduction in VAS-A scores after exposure to Hindu spiritual music in elderly patients [8]. However, unlike our study, which compared spiritual music with non-spiritual music, their study used a control group with no music.

Our study found a significant reduction in serum cortisol levels in the spiritual music group, indicating a lower neuroendocrine stress response. Serum cortisol is a well-established marker of the endocrine stress response to surgery and was used in this study as a biochemical indicator of stress. Anxiety and stress are closely linked, with elevated anxiety levels often leading to an increased physiological stress response, including activation of the hypothalamic-pituitary-adrenal (HPA) axis and subsequent cortisol release [20,21]. Our findings are consistent with Leardi et al., who compared researcher-selected new-age music with subject-selected music and found that postoperative cortisol levels were significantly lower in the group that listened to their preferred music [22]. The reduction in stress and anxiety observed with spirituality-based interventions can be attributed to several physiological mechanisms. Spiritual engagement has been shown to downregulate the hypothalamic-pituitary-adrenal (HPA) axis, leading to decreased cortisol secretion and a reduced physiological stress response [23]. In addition, meditative and spiritual practices enhance prefrontal cortex activity, which plays a crucial role in emotional regulation while simultaneously reducing amygdala hyperactivity, thereby dampening the brain's stress and fear responses [24]. Furthermore, spiritual practices such as prayer, meditation, and chanting stimulate the parasympathetic nervous system, resulting in lower heart rate, blood pressure, and cortisol levels, which contribute to a state of relaxation [25]. These neurophysiological effects provide a strong basis for the role of spirituality in stress reduction.

We found higher patient satisfaction in the spiritual music group, suggesting that music aligned with personal beliefs may enhance emotional comfort in perioperative settings. No previous study has directly compared patient satisfaction between spiritual and non-spiritual music in a surgical setting.

This study has several limitations. First, the study population included patients undergoing lower limb surgery alone, which may limit the generalizability of the findings to other surgical procedures. Second, the sample size was relatively small, which may affect the statistical power and broader applicability of the results. Third, stress response was evaluated solely using serum cortisol levels, and other potential physiological or biochemical markers of stress were not assessed. Lastly, the study focused primarily on anxiety and stress response, without assessing other cognitive domains, such as emotional resilience, coping mechanisms, or long-term psychological effects of spiritual music.

To the best of our knowledge, this is the first study to directly compare Hindu spiritual music with non-spiritual music in a randomized controlled trial for perioperative anxiety management. While the role of music therapy in reducing stress and anxiety has been explored in various clinical settings, prior studies have primarily compared spiritual or non-spiritual music with no music rather than evaluating their relative efficacy. The strength of our study lies in its patient-centered approach, allowing participants to select their preferred music within their assigned category, thereby reducing observer bias and ensuring a more personalized intervention. Unlike previous research where music was often preselected by the investigators, our methodology ensured that cultural relevance and personal preference were accounted for, which may have contributed to the greater reduction in anxiety and stress response observed in the spiritual music group. These findings highlight the potential for integrating faith-based interventions into clinical practice to enhance perioperative care.

## Conclusions

Hindu spiritual music effectively reduces perioperative anxiety and stress response in patients undergoing lower limb surgery under regional anesthesia. It also decreases the incidence of postoperative nausea and vomiting while improving patient satisfaction. Given its non-invasive and culturally acceptable nature, it should be considered a viable non-pharmacological intervention for perioperative anxiety management. Future research should explore whether similar benefits are observed in non-Hindu religious individuals listening to music or hymns of their own faith and cultural background.

## Figures and Tables

**Figure 3:**
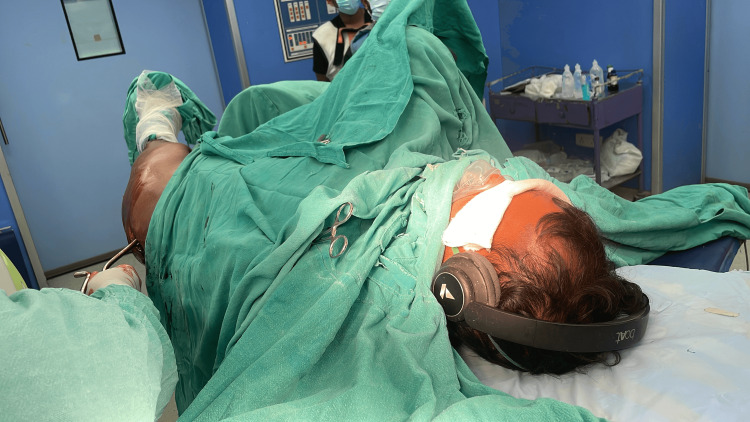
Intraoperative application of music intervention using headphones in a patient undergoing lower limb surgery under regional anesthesia

## Appendices

Appendix A

**Table 5:** Hindu religious songs (calm and rhythmic versions) used for spiritual music intervention in the study Footnote: Subjects could deselect two songs from this list based on their preference.

S. No.	Song name	Duration (seconds)
1	Om Gan Ganapataye Namah (9 times)	184
2	Twam-eva Mata (9 times)	132
3	Gayatri Mantra (9 times)	128
4	Raghupati Raghav Raja Ram	202
5	Maha Mrityunjaya Mantra (9 times)	132
6	Om Jai Jagdish Hare	361
7	Hanuman Chalisa (slow)	461
8	Sri Ramchandra Kripalu	284
9	Achyutam Keshavam	346
10	Hare Rama Hare Krishna	410

Appendix B
